# Mental Health Day Hospitals and Lockdown Due to COVID-19 in Spain

**DOI:** 10.3389/fpsyg.2022.769015

**Published:** 2022-03-10

**Authors:** Antonio José Sánchez-Guarnido, Valentina Lucena, Aurora Torrent, Laura Bosa, Virginia Martinez-López, Ana Cuartiles-Berenguer, Iolanda Batalla

**Affiliations:** ^1^Santa Ana Hospital, Granada, Spain; ^2^Department of Psychology, Córdoba University, Córdoba, Spain; ^3^Santa Maria University Hospital, Lleida, Spain

**Keywords:** mental health, day hospital, COVID-19, social distancing, compliance

## Abstract

**Introduction:**

The COVID-19 pandemic has forced changes in patient care in Mental Health Day Hospitals (MHDHs).

**Objectives:**

To study the interventions performed in MHDHs during the pandemic in comparison with those performed in other facilities and to analyze the new hospital admissions in both groups.

**Method:**

A retrospective multicenter cohort study comparing the interventions received by a group of 161 patients admitted in MHDHs during the lockdown period in Spain with the interventions of another group of 109 patients who were treated at other facilities during lockdown.

**Results:**

MHDHs reduced their face-to-face interventions during lockdown just as much as other facilities but implemented telematic intervention methods to a greater extent. Patients attached to MHDHs during lockdown were admitted significantly less and presented fewer urgent consultations in the following 6 months.

**Conclusion:**

The use of telepsychiatry made it feasible to adapt MHDHs to periods of lockdown, being useful to improve the continuity of care during the pandemic. In addition, it was possible to maintain a reduction in hospital admissions in patients treated at MHDHs.

## Introduction

On March 14, 2020, a state of alarm was declared in Spain [Boletín Oficial del Estado (Official Spanish Government Gazette), 2020], with people being confined to their homes because of the COVID-19 pandemic. Social isolation subsequent to this lockdown generated psychological distress in the general population (Parrado-González and León-Jariego, 2020) and, to a greater extent, in patients with severe mental disorders, who had less healthy behavior strategies to cope with the situation (Hao et al., 2020; Barlati et al., 2021; Solé et al., 2021).

During this period, psychiatric facilities had to reorganize their care in a matter of days (Moreno et al., 2020), promoting telematic care, or telepsychiatry (Gentile et al., 2020). Some mental health day hospitals (MHDHs) were closed (Vieta et al., 2020), leaving severe patients without follow-up. Others had to resort to telematic interventions to be able to continue to offer optimal, individualized care for each patient during lockdown (de Brouwer et al., 2020; Koreki et al., 2020; Salles et al., 2020). This new form of communication provided a useful channel of intervention for patients, with instant, continuously updated follow-up (Gentile et al., 2020). Telepsychiatry has definitely begun to be implemented in MHDHs and, since this technology is currently used by 57% of the population and is expected to reach 90% by 2030 (Gentile et al., 2020), it may well become a permanent feature of these units in the near future.

Research into telepsychiatry has in general produced positive results in terms of acceptance and satisfaction levels among both patients and health professionals. The viability and efficiency of such treatment have been proven (Cowan et al., 2019; Smith et al., 2020). Evidence has also been found regarding the use of different psychotherapeutic models such as cognitive behavioral therapy (Etzelmueller et al., 2018), dialectic behavior therapy (Oliveira and Rizvi, 2018), interpersonal therapy (Dennis et al., 2020), and psychoanalysis (Bakalar, 2013). Telepsychiatry has been proven to be efficient for the treatment of different disorders, including anxiety, depression, post-traumatic stress, and eating disorders (Mitchell et al., 2008; Poletti et al., 2020).

However, telepsychiatry also raises a number of concerns, the risk of deterioration in the therapeutic relationship, teamwork, confidentiality, and privacy, for example (Cowan et al., 2019; Smith et al., 2020). Furthermore, despite the reported benefits of treating different pathologies telematically, it is an area that has not yet been studied in any depth in people with severe mental disorders (SMD; Santesteban-Echarri et al., 2018).

Several studies have demonstrated the efficacy of MHDHs in the clinical improvement and stability of patients admitted to such units (Marshall et al., 2001a,b; Duarte et al., 2019), reporting a decrease in relapses in the form of lower numbers of hospital readmissions and visits to emergency departments (Quesada Franco et al., 2006; Duarte et al., 2019; Heekeren et al., 2020). However, there is no evidence about how these facilities have performed during the pandemic or whether they have been able to maintain their level of effectiveness.

The aim of this study was therefore to test whether the implementation of telematic tools in MHDHs effectively reduces the number of visits to the emergency or hospital admissions in the context of the decline in face-to-face interventions brought about by the COVID-19 pandemic.

## Materials and Methods

### Design

A multicenter retrospective cohort study.

### Participants

The study was carried out with people over 18 years of age, of both sexes, who had been under follow-up in MHDHs at some time during the year 2020. Fifteen hospitals in Spain participated and data were collected from a total of 270 patients: 120 men and 150 women, all aged between 18 and 67 years (average age 39.90). The most common diagnoses were psychotic disorders (30.4%), personality disorders (27.8%), and bipolar disorder (10.4%). Most of the participants had primary (35.6%) or secondary (41.5%) levels of education, while 14.8% had studied at university level. They lived with their family of origin (28.9%), in their own family home (28.9%), or alone (17%). A total of 29.3% were retired, 26.3% were unemployed, 20% were temporarily unable to work due to disability, and 16.7% were working.

### Procedure

Interventions in MHDHs during the lockdown period were received by 161 patients while 109 patients were treated in other facilities in that same period (56 in community mental health units, 17 in primary care, 2 in the therapeutic community, and 34 in other facilities).

The data were collected retrospectively during the months of October and November 2020 by the collaborators at each MHDH, from the patients’ clinical histories. To guarantee capacity, coherence, and correctness in the collection of data from the 15 centers, three online training sessions were held for the professionals involved. A password-protected database was designed and clinical data were processed without patient identification data.

### Measures

The sociodemographic variables studied were age, sex, composition of the household where the patient lived, employment status, and level of education.

Having been treated in an MHDH during the period of lockdown following the outbreak of the COVID-19 pandemic in Spain (March 16–May 15) was used as an independent variable.

Psychotherapy, occupational therapy, and nursing interventions received face-to-face, by telephone, by videoconference, or by other telematic means (WhatsApp, Facebook, blog, or email) were studied as dependent variables. This information was coded dichotomously according to whether or not the different forms of treatment were received during this period.

To compare relapse rates, full hospital admissions at two, four, and 6 months after lockdown were collected (dichotomously: admission/non-admission), and the mean number of emergencies per patient at two, four, and 6 months was used as a secondary response variable.

### Data Analysis

The data were analyzed statistically using the IBM-SPSS V.21.0 program and the level of statistical significance used was *p* < 0.05.

#### Descriptive Statistics

The results of the categorical variables were expressed as percentages and those of the quantitative variables as mean and standard deviation.

#### Bivariate Analysis

Chi-square was used in the between-group analyses to analyze categorical variables and Student’s *t*-test was used to compare quantitative variables.

## Results

### Analysis of the Two Groups’ Sociodemographic and Clinical Characteristics

There were no differences between the two groups in sex (*χ*^2^ = 1.685; *p* = 0.194), age (*t* = 1.207; *p* = 0.149), household composition (*χ*^2^ = 2.843; *p* = 0.416), educational level (*χ*^2^ = 4.045; *p* = 0.400; Table 1), or use of long-acting injectable (LAI) antipsychotics (*χ*^2^ = 2.248; *p* = 0.134), adherence to treatment (*χ*^2^ = 0.889; *p* = 0.346). There were significant differences in employment, with only 10.6% of the MHDH patients working compared to 25.7% of the group treated in other facilities (*χ*^2^ = 12.850; *p* = 0.025). The most common diagnoses in both groups were schizophrenia and other psychotic disorders, followed by personality disorders (*χ*^2^ = 13.88; *p* = 0.053).

**Table 1:** Sociodemographic and clinical characteristics.

		Day Hospital	Other facilities		
		*N* = 161	*N* = 109		
Variable	Category			*X^2^ or t*	*p*
Gender	Female	84(52.2%)	65(60.2%)	1.685	0.194
	Male	77(47.8%)	43(39.8%)		
Age Half (DE)		39,19(11,37)	40,95(12,40)	*t* = 1.207	0.149
Diagnosis	Schizophrenia or other psychotic disorders	54(33.5%)	28(25.7%)	8.177	0.085
	Bipolar disorder	16(9.9%)	12(11%)		
	Personality disorder	48(29.8%)	27(24.8%)		
	Major depressive disorder	17(10.6%)	9(8.3%)		
	Other	26(16.1%)	33(30.3%)		
Household composition	Complete family or origin	75(46.6%)	40(37%)	2.843	0.416
	Own family home	41(25.5%)	36(33.3%)		
	Sole proprietorship	27(16.8%)	19(17.6%)		
	Other	18(11.2%)	13(12.0%)		
Activity	Work/Vocational/ Occupational Activity before the pandemic				
	Retired, pensioner	52(32.3%)	27(24.8%)	12.850	0.025^*^
	Unemployed	48(29.8%)	23(21.1%)		
	Working	17(10.6%)	28(25.7%)		
	Student	12(7.5%)	8(7.3%)		
	Volunteer/Mutual Aid Agent	1(0.6%)	0(0%)		
Education level	Without studies	3(1.9%)	5(4.6%)	4.045	0.400
	Primary	62(38.5%)	34(31.2%)		
	Secondary	68(42.2%)	44(40.4%)		
	University	7(4.3%)	7(6.4%)		
Prescription of long-acting injectable (LAI)	LAI	30(18.6%)	13(11.9%)	2.248	0.134
Correct adherence to pharmacological treatment	Takes medication correctly	141(87.6%)	91(83.5%)	0.889	0.346

* *p < 0.05.*

### Analysis of the Interventions Received

Table 2 shows the differences between the psychotherapeutic, occupational therapy, and nursing interventions received face-to-face or online by patients treated in MHDHs during lockdown and those received by patients treated in other facilities. As can be seen, there were no significant differences in the face-to-face interventions in either psychotherapy (*p* = 0.243), occupational therapy (*p* = 0.744), or nursing (*p* = 0.679). Significant differences were, however, found in all online psychotherapy, occupational therapy, and nursing interventions in MHDHs. Telephone interventions differed significantly in psychotherapy (77.6% vs. 33.6%; *p* < 0.001), occupational therapy (34.2% vs. 0.9%; *p* < 0.001), and nursing (46% vs. 14.7%; *p* < 0.001). The numbers of videoconference interventions were higher in MHDHs in psychotherapy (16.1% vs. 2%; *p* < 0.001), occupational therapy (14.3% vs. 0.9%; *p* < 0.001), and nursing (14.3% vs. 0.9%; *p* < 0.001). The use of other telematic tools was also greater in MHDHs in psychotherapy (11.2% vs. 0%; *p* < 0.001), occupational therapy (44.7% vs. 4.6%; *p* < 0.001), and nursing (21.1% vs. 0%; *p* < 0.001).

**Table 2:** Differences between the psychotherapeutic, occupational therapy, and nursing interventions received face-to-face, by telephone, by videoconference, or by other telematic means by patients treated in MHDHs during lockdown and those received by patients in other facilities.

	Day Hospital (*N* = 161)	Other (*N* = 109)	*X^2^*	*p*
*Psychotherapy*				
Face-to-face	41(25.5%)	21(19.3%)	1.43	0.243
By telephone	125(77.6%)	37(33.6%)	52.64	0.000^*^
By videoconference	26(16.1%)	3(2%)	14.33	0.000^*^
Other	18(11.2%)	0(0%)	19.47	0.000^*^
*Occupational Therapy*				
Face-to-face	12(7.5%)	7(6.4%)	0.107	0.744
By telephone	55(34.2%)	1(0.9%)	57.54	0.000^*^
By videoconference	23(14.3%)	1(0.9%)	18.54	0.000^*^
Other	72(44.7%)	5(4.6%)	60.82	0.000^*^
*Nursing*				
Face-to-face	25(15.5%)	19(17.4%)	0.17	0.679
By telephone	74(46%)	16(14.7%)	30.64	0.000^*^
By videoconference	23(14.3%)	1(0.9%)	18.54	0.000^*^
Other	34(21.1%)	0(0%)	38.43	0.000^*^

*p < 0.001.

### Analysis of Hospital Admissions and Emergency Consultations

In relation to hospital admissions, significantly lower percentages of admissions were found in patients receiving care from MHDHs during lockdown at 2 months (1.9% vs. 13.8%; *p* < 0.001), at 4 months (5.6% vs. 25.7%; *p* < 0.001), and at 6 months (9.9% vs. 35.8%; *p* < 0.001; see the online supplement to this report). In terms of emergency consultations, patients who were at MHDHs during lockdown had on average 0.327 fewer emergencies at 2 months (*t* = 2.54; *d* = 0.38 *p* = 0.001), 0.881 fewer emergencies at 4 months (*t* = 2.67; *d* = 0.39; *p* < 0.001), and 1.067 fewer emergencies at 6 months (*t* = 2.14; *d* = 0.30; *p* = 0.003; Table 3).

**Table 3:** Comparison of admissions and emergencies among patients attended in DHs during lockdown and those attended in other facilities.

		Day Hospital (*N* = 161)	Other (*N* = 109)	*X^2^ or t*	*p*
Hospital admissions (percentages and chi-square)	2 months after the periodic of confinement	3(1.9%)	15(13.8%)	15.08	0.000^*^
	4 months after the periodic of confinement	9(5.6%)	28(25.7%)	22.14	0.000^*^
	6 months after the periodic of confinement	16(9.9%)	39(35.8%)	26.56	0.000^*^
Emergency consultations (Mean, SD, and Student’s *t*-test)	2 months after the periodic of confinement	0,13(0.46)	0,69(2.26)	2.54	0.000^*^
	4 months after the periodic of confinement	0,32(1.24)	1,22(3.32)	2.67	0.000^*^
	6 months after the periodic of confinement	0,6(2,19)	1,68(4,95)	2.14	0.000^*^

* *p < 0.001.*

## Discussion

The aim of this article is to study the interventions carried out in MHDHs during the pandemic in comparison with those carried out in other facilities and to determine whether the effectiveness of these units in preventing relapses was maintained during this period.

The results obtained confirm that, compared to other facilities, MHDHs adapted the follow-up of their patients more successfully to the limitations of lockdown by introducing and/or increasing telecare. A study conducted during lockdown in a Japanese day hospital (Koreki et al., 2020) reports that weekly telephone monitoring was able to help maintain routines and minimize the effects of uncertainty and unpredictability. We are not aware of any other studies that provide objective data on the impact of such adaptation in psychiatric day hospitals.

Our study demonstrates that such adaptation has made it possible to maintain the efficacy of mental healthcare in terms of hospital admissions and emergency department care. During lockdown, the number of psychiatric emergencies generally decreased (Pacchiarotti et al., 2020; Rodriguez et al., 2021) and the proportion of hospital admissions in patients attending emergency departments increased due to higher levels of clinical severity (Rodriguez et al., 2021). These figures returned to normal levels over the following months (Rodriguez et al., 2021). The continuity of care in day hospitals decreased admissions and the number of patients being treated in these facilities.

The research presented in this article opens the door to further study into changes in the interventions carried out in MHDHs during the pandemic, an issue which has scarcely been addressed in the scientific literature. Its descriptive observational approach throws light on the actual practices developed in the COVID-19 situation. Albeit retrospectively, the study allowed us to compare those changes in relation both to the inclusion of different channels of intervention and to the results in terms of relapses. The fact that it is a multicenter study also facilitates greater generalization of the results obtained. We know that during the first few months of the pandemic, it was assumed that face-to-face interventions would increase the risk of infection among patients and health professionals, and this led to major changes in mental healthcare. In October 2020, the WHO reported that around 40% of all psychosocial and psychotherapeutic interventions in Europe had been brought to a halt since the beginning of the crisis. This represented a challenge for programs providing services to SMD patients (Kozloff et al., 2020).

As can be seen in our results, telephone and videoconference consultations were implemented to alleviate this disconnection. But this increased use of telematic healthcare gave rise to a series of fears regarding possible negative effects on the therapeutic relationship, confidentiality, and privacy (Cowan et al., 2019; Smith et al., 2020). It is also necessary to train health professionals in the skills needed for this form of therapy (Cowan et al., 2019; Smith et al., 2020) and ensure that patients have access to the corresponding telematic resources (Smith et al., 2020). We also know that people suffering from psychosis tend to use digital technology less (Robotham et al., 2016). Despite all this, however, current evidence shows that telehealth can contribute to improvements in patients’ conditions, even though more data are required in this respect (Kasckow et al., 2014). It would be useful also to analyze the limitations of existing research and the risks of using this technology (López-Santín and Serón, 2018).

### Limitations and Future Directions

This study has methodological limitations that should be taken into account. Being an observational study, it is not possible to establish causal relationships, and we assume that some of the associations found may be contaminated or interact with other variables not studied. Given the characteristics of the period studied, however, any other type of study would be impossible. The fact that this is a retrospective study may also introduce some biases, although objective variables based on medical records were used to reduce them. The other resources sampled are heterogeneous and sometimes limited in number. This must be taken into account when interpreting the results.

We believe that these limitations do not diminish the importance of the results, but it would be interesting to carry out prospective studies with a larger number of patients in different psychiatric facilities in order to confirm that the switch to telepsychiatry in day hospitals maintains their efficacy compared to other units. It may also be of interest to obtain more information about the specific interventions carried out, their frequency and the individual or group format used. It is important to add other measures for outcome assessment based on psychopathological, functional, and recovery models, and to incorporate an analysis segregated according to different diagnoses. It would also be of interest to keep monitoring patients in the months following changes in intervention pathways. Finally, we believe that more research is required in order to confirm the efficacy of using telematic channels of intervention once the pandemic has passed. Such research could, for example, look at mixed models of healthcare (face-to-face and online) adapted to the needs and possibilities of each patient.

## Conclusion

In summary, these results support the feasibility of adapting the functioning of MHDHs to the constraints of lockdown periods by using telepsychiatry and of preserving these services in order to maintain their effectiveness. With this in mind, day hospitals should be provided with sufficient telematic resources to be able to address similar situations in the future.

## Data Availability Statement

The raw data supporting the conclusions of this article will be made available by the authors, without undue reservation.

## Ethics Statement

The studies involving human participants were reviewed and approved by the research ethics committees of the different participating hospitals. The principles of the Helsinki Declaration were complied with at all times. Data confidentiality was respected in accordance with the latest European Union (EU) legislation. The patients/participants provided their written informed consent to participate in this study.

## Author Contributions

AS-G contributed to conception and design of the study. AT, VM-L, and AC-B organized the database. VL performed the statistical analysis and wrote sections of the manuscript. AS-G and IB wrote the first draft of the manuscript. All authors contributed to the article and approved the submitted version.

## Funding

The research was funded under the FPS 2020—R&I call for projects in primary care, county hospitals, and CHARES, under project code AP-0028-2020-C1-F2. Fundación Pública Andaluza para la Investigación Biosanitaria de Andalucía Oriental.

## Conflict of Interest

The authors declare that the research was conducted in the absence of any commercial or financial relationships that could be construed as a potential conflict of interest.

## Publisher’s Note

All claims expressed in this article are solely those of the authors and do not necessarily represent those of their affiliated organizations, or those of the publisher, the editors and the reviewers. Any product that may be evaluated in this article, or claim that may be made by its manufacturer, is not guaranteed or endorsed by the publisher.
